# *L*(2,1)-Labeling of the Strong Product of Paths and Cycles

**DOI:** 10.1155/2014/741932

**Published:** 2014-02-24

**Authors:** Zehui Shao, Aleksander Vesel

**Affiliations:** ^1^School of Information Science and Technology, Chengdu University, Chengdu 610106, China; ^2^Key Laboratory of Pattern Recognition and Intelligent Information Processing, Institutions of Higher Education of Sichuan Province, Sichuan 610106, China; ^3^Faculty of Natural Sciences and Mathematics, University of Maribor, Koroška Cesta 160, 2000 Maribor, Slovenia

## Abstract

An *L*(2,1)-labeling of a graph *G* = (*V*, *E*) is a function *f* from the vertex set *V*(*G*) to the set of nonnegative integers such that the labels on adjacent vertices differ by at least two and the labels on vertices at distance two differ by at least one. The span of *f* is the difference between the largest and the smallest numbers in *f*(*V*). The *λ*-number of *G*, denoted by *λ*(*G*), is the minimum span over all *L*(2,1)-labelings of *G*. We consider the *λ*-number of *P*_*n*_⊠*C*_*m*_ and for *n* ≤ 11 the *λ*-number of *C*_*n*_⊠*C*_*m*_. We determine *λ*-numbers of graphs of interest with the exception of a finite number of graphs and we improve the bounds on the *λ*-number of *C*_*n*_⊠*C*_*m*_, *m* ≥ 24 and *n* ≥ 26.

## 1. Introduction

The Frequency Assignment Problem (FAP) requires assigning frequencies to transmitters in a wireless network. In a broadcasting network, each transmitter is assigned a frequency channel for its transmissions. Two transmissions can interfere if their channels are too close. That means that even if two transmitters use different channels, there still may be interference if the two transmitters are located close to each other [1, 2].

The spectrum of frequencies gets more and more scarce because of increasing demands, both civil and military. Thus the task is to minimize the span of frequencies while avoiding interference. One of the graph-theoretical models of FAP which is well elaborated is the concept of distance-constrained labeling of graphs [1]. Many variants of this concept have been proposed; however, the *L*(2,1)-labeling problem where adjacent vertices must be assigned colors of distance at least two apart and vertices of distance two must be assigned different colors has attracted the most of interest [3, 4].

An *L*(2,1)-labeling of a graph *G* is a function *f* from the vertex set *V*(*G*) to the set of nonnegative integers *C* (called *labels* or *colors*) such that for any two vertices *u* and *v*

(1) $$\begin{array}{c} \left|f\left(u\right)-f\left(v\right)\right|\geq 2\;\text{if}\;\;d\left(u,v\right)=1,\\ \left|f\left(u\right)-f\left(v\right)\right|\geq 1\;\text{if}\;\;d\left(u,v\right)=2. \end{array}$$

A *k*-*L*(2,1)-*labeling* is a *L*(2,1)-labeling of *G* such that *C* = {0,…, *k*}. An optimal *L*(2,1)-labeling of *G* is a *k*-*L*(2,1)-labeling with *k* smallest possible. The largest label used by an optimal *L*(2,1)-labeling is called the *λ*-*number *of *G* and denoted by *λ*(*G*).

There is a number of studies on the algorithms for *L*(2,1)-labeling problem [1, 5, 6]. It is known to be **NP**-hard for general graphs [4]. Even for some relatively simple families of graphs such as planar graphs, bipartite graphs, chordal graphs [5], and graphs of treewidth two [7], the problem is also **NP**-hard.

Product graphs are considered in order to gain global information from the factor graphs [8]. Many interesting wireless networks are based on product graphs with simple factors, such as paths and cycles. In particular, any square grid (resp., torus) is the Cartesian product of two paths (resp., cycles) and any octagonal grid (resp., torus) is the strong product of two paths (resp., cycles) [9]. For the Cartesian product of these factors the *λ* numbers have been completely determined [10–12], while for the strong and the direct product only partial results have been found [13–15].

The paper is organized as follows. In Section 2, we give definitions and concepts needed in this paper. We also report on the known results for the *λ* numbers of the graphs of interest. In Section 3, two main computer search methods applied in the paper are described: the dynamic algorithm and the SAT reduction. Finally, in Section 4, we present the results on the *λ*-number of *P*_*n*_⊠*C*_*m*_ and the *λ*-number of *C*_*n*_⊠*C*_*m*_.

## 2. Preliminaries and Previous Results

For a graph *G* = (*V*, *E*), *V*(*G*) and *E*(*G*) are the sets of vertices and edges of *G*, respectively. A directed graph *D* consists of vertices *V*(*D*) together with a set of arcs *A*(*D*)⊆*V*(*D*) × *V*(*D*). We write *G* also to stand for the vertex set of the graph *G*. In this paper, only directed and undirected graphs without multiple edges or loops are considered.

The *strong product* of graphs *G* and *H* is the graph *G*⊠*H* with vertex set *G* × *H* and (*x*_1_, *x*_2_)(*y*_1_, *y*_2_) ∈ *E*(*G*⊠*H*) whenever *x*_1_ *y*_1_ ∈ *E*(*G*) and *x*_2_ = *y*_2_, or *x*_2_ *y*_2_ ∈ *E*(*H*) and *x*_1_ = *y*_1_, or *x*_1_ *y*_1_ ∈ *E*(*G*) and *x*_2_ *y*_2_ ∈ *E*(*H*). The strong product is commutative and associative, having the trivial graph as a unit (cf. [8]). The subgraph of *G*⊠*H* induced by *u* × *V*(*H*) is isomorphic to *H*. It is called an *H*-fiber and denoted by *H* ^*u*^.

**Figure 1:**
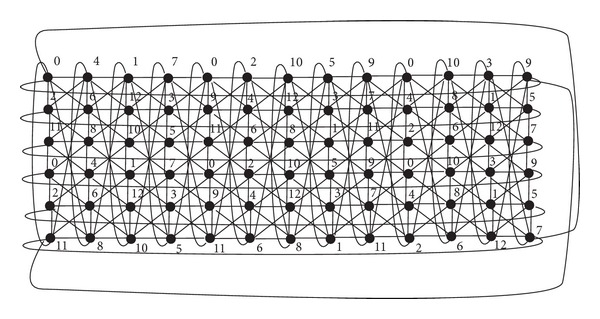
Strong product *C*_6_⊠*C*_13_ with a 12-*L*(2,1)-labeling.

The *path P*_*n*_ is the graph whose vertices are 0, 1,…, *n* − 1 and for which two vertices are adjacent precisely if their difference is ±1. For an integer *n* ≥ 3, the *cycle of length n* is the graph *C*_*n*_ whose vertices are 0, 1,…, *n* − 1 and whose edges are the pairs *i*, *i* + 1, where the arithmetic is done modulo *n*. Note that the strong product *C*_6_⊠*C*_13_, depicted in Figure 1, can be regarded as a graph composed of six copies of *C*_13_ (denoted by *C*_13_^0^,…, *C*_13_^5^) or a graph composed of 13 copies of *C*_6_ (denoted by *C*_6_^0^,…, *C*_6_^12^).

A *walk* in a directed graph *D* is a sequence of (not necessarily distinct) vertices *v*_1_,*v*_2_,…, *v*_*n*_ such that *v*_*i*_ *v*_*i*+1_ ∈ *A*(*D*) for 1, 2,…, *n* − 1. If *v*_1_ = *v*_*n*_, we say it is a *closed walk*.

If *P* is a path (resp., walk), then its *length* is its number of edges (resp., arcs).

The following simple lemma is well known.

**Lemma 1**

If *H* is a subgraph of *G*, then *λ*(*H*) ≤ *λ*(*G*).

Let *f* denote a *k*-*L*(2,1)-labeling of *C*_*n*_⊠*G*. We denote by *f*_*i*,*p*_ the restriction of *f* to *G* ^*i*^, *G* ^*i*+1^,…, *G* ^*i*+*p*−1^, *i* = 0,1,…, *n* − 1 and *p* = 1,…, *n* − *p*. Note that *G* ^*i*^ is isomorphic to *G*; that is, *G* ^*i*^ is the subgraph of *G*⊠*C*_*n*_ induced by *V*(*G*) × *i*. We will also write *f*_*i*_ for *f*_*i*,1_.

The following lemma provides an upper bound for the *λ*-number of the strong product of a graph with a cycle.

**Lemma 2**

Let *t* ≥ 1 and *f* be a *k*-*L*(2,1)-labeling of *C*_*n*_⊠*G*. If *f*_0,*p*_ is a *k*-*L*(2,1)-labeling of *C*_*p*_⊠*G*, then *λ*(*C*_*n*+(*t*−1)*p*_⊠*G*) ≤ *k*.

**Proof**

Let *f*′ be a function from *V*(*C*_*n*+(*t*−1)*p*_⊠*G*) onto the set {0,1,…, *k*} and *f*_*i*_′ the restriction of *f*′ to *V*(*G* ^*i*^). The function *f*′ is defined as follows:

(2) $$\begin{array}{c} f_{i}^{\prime }=\{\begin{array}{cc} f_{i}, & i<n\\ f_{(i-n)\;\mathrm{mod}\;p} & i\geq n. \end{array} \end{array}$$

It is not difficult to see that *f*′ is a *k*-*L*(2,1)-labeling of *C*_*n*+(*t*−1)*p*_⊠*G*.

Given two integers *r* and *s*, let *S*(*r*, *s*) denote the set of all nonnegative integer combinations of *r* and *s*:

(3) $$\begin{array}{c} S\left(r,s\right)=\left\{\alpha r+\beta s:\alpha ,\;\;\beta \in Z^{+}\right\}. \end{array}$$

We will need the result of Sylvester [16].

**Lemma 3**

If *r*, *s* > 1 are relatively prime integers, then *t* ∈ *S*(*r*, *s*) for all *t* ≥ (*s* − 1)(*r* − 1).

Some partial results on the *λ*-number for the strong products of two cycles are given in [15].

**Theorem 4**

Let *m* ≥ 3. Then

(4) $$\begin{array}{c} \lambda \left(C_{3}⊠C_{m}\right)=\{\begin{array}{cc} 16, & m=3,6\\ 14, & m=5,7,10,11,15\\ 13, & m=9,14,18,19,22,23,27,31,35\\ 12, & otherwise. \end{array} \end{array}$$

**Theorem 5**

Let *m* ≥ 3. Then

(5) $$\begin{array}{c} \lambda \left(C_{4}⊠C_{m}\right)=\{\begin{array}{cc} 19, & m=5\\ 15, & m=4,8\\ 14, & m=11\\ 13, & m=7,10,14,17,20,23\\ 11, & m\equiv 0\;\left(\mathrm{mod}\;6\right)\\ 12, & otherwise. \end{array} \end{array}$$

**Theorem 6**

If *m* ≥ 24 and *n* ≥ 36, then *λ*(*C*_*n*_⊠*C*_*m*_) ≤ 12.

For the strong product of more than two cycles the following result presented in [14] is known.

**Theorem 7**

If *k* ≥ 1 and *m*_0_,…, *m*_*k*−1_ are each multiple of 3^*k*^ + 2, then *λ*(*C*_*m*_0__⊠⋯⊠*C*_*m*_*k*−1__) = 3^*k*^ + 1.

## 3. Computer Search

### 3.1. Dynamic Algorithm

The idea is introduced in [10] in a more general framework and later used several times, for example, [13, 15]. In order to make the paper self-contained we first describe its basic definitions and results.

We define a digraph *D*_*n*,*k*_ as follows. Its vertices are the *k*-*L*(2,1)-labelings of *C*_*n*_⊠*P*_2_. Let *u* = *u*_1_ *u*_2_ be a vertex of *D*_*n*,*k*_. Then *u*_1_ and *u*_2_ represent the *k*-*L*(2,1)-labeling of *C*_*n*_⊠*P*_2_ restricted to the first and second copies of *C*_*n*_, respectively.

Let *u* and *v* be two vertices of *D*_*n*,*k*_. Then $\tilde{uv}$ denotes the labeling of *C*_*n*_⊠*P*_3_ obtained by applying *u*_1_, *u*_2_, and *v*_2_ to the consecutive copies of *C*_*n*_. (Note that $\tilde{uv}$ is not always a *k*-*L*(2,1)-labeling of *C*_*n*_⊠*P*_3_.) We make an arc from *u* to *v* in *D*_*n*,*k*_ if and only if the following two conditions are fulfilled:

(i) *u*_2_ equals *v*_1_; (*ii*) $\tilde{uv}$ is a *k*-*L*(2,1)-labeling of *C*_*n*_⊠*P*_3_.

Analogously, we define a digraph *D*_*n*,*k*_′ with the vertex set composed by *k*-*L*(2,1)-labelings of *P*_*n*_⊠*P*_2_. In other words, if *u* = *u*_1_ *u*_2_ is a vertex of *D*_*n*,*k*_′, then *u*_1_ and *u*_2_ represent the *k*-*L*(2,1)-labeling of *P*_*n*_⊠*P*_2_ restricted to the first and second copies of *P*_*n*_, respectively. The set of arcs of *D*_*n*,*k*_′ is formed analogously as the set of arcs of *D*_*n*,*k*_.

**Figure 2:**
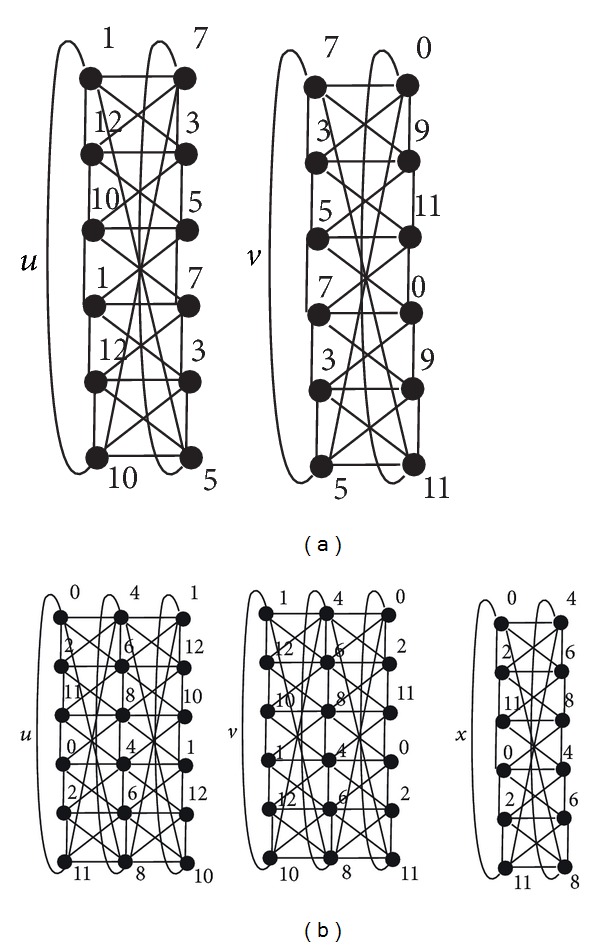
Two vertices of *D*_6,12_ (a) and two vertices of *V*_6,12_^3^ with a vertex of *D*_6,12_^2^ (b).

Figure 2(a) shows two vertices of *D*_6,12_ denoted by *u* and *v*. We can see that the labeling of the second copy of *C*_6_ in *u* equals the labeling of the first copy of *C*_6_ in *v*. Moreover, the labeling of *u* and the labeling of the second copy of *C*_6_ in *v* induce a 12-*L*(2,1)-labeling of *C*_6_⊠*P*_3_. It follows that *A*(*D*_6,12_) admits an arc from *u* to *v*.

The next theorem follows from the results presented in [10].

**Theorem 8**

*C*_*i*_⊠*C*_*ℓ*_ (resp., *P*_*i*_⊠*C*_*ℓ*_) admits a *k*-*L*(2,1)-labeling if and only if *D*_*i*,*k*_ (resp. *D*_*i*,*k*_′) contains a closed directed walk of length *ℓ*.

The dynamic algorithm first generates all *k*-*L*(2,1)-labelings of *C*_*i*_⊠*P*_2_ which are the vertices of *D*_*i*,*k*_. Since a main building block *C*_*i*_⊠*P*_2_ is usually relatively small, a simple method, for example, backtracking, can be applied for this step. In the next step, the set of edges of *D*_*i*,*k*_ has to be generated. The procedure for this step is described in [15]. The described algorithm however has the time complexity *O*(*n* ^2^), where *n* denotes the number of vertices in *D*_*i*,*k*_. Note that *n* can be very large even for *i* and *k* of a moderate size. Some examples for *i* and *k* of interest are |*D*_5,14_| = 114984000, |*D*_6,11_| = 1925760, and |*D*_8,11_| = 1072523264. The complexity of the algorithm does not allow a computation of *A*(*D*_*i*,*k*_) in a reasonable time for these cases. We have therefore improved this method as described in the sequel.

Let *V*_*i*,*k*_^3^ denote the set of all *k*-*L*(2,1)-labelings of *C*_*i*_⊠*P*_3_. If *u* is an element of *V*_*i*,*k*_^3^, then *u*_1_, *u*_2_, *u*_3_ denote the restriction of *u* to the first, second, and third copies of *C*_*i*_ in *C*_*i*_⊠*P*_3_; respectively. Note that *V*_*i*,*k*_^3^ contains symmetric labelings of *C*_*i*_⊠*P*_3_, that is, if *u* ∈ *V*_*i*,*k*_^3^, then *v* ∈ *V*_*i*,*k*_^3^ exists, such that *u*_1_ = *v*_3_, *u*_3_ = *v*_1_, and *u*_2_ = *v*_2_.

We now define the digraph *D*_*i*,*k*_^2^ as follows. Let *V*(*D*_*i*,*k*_^2^) denote the set of *k*-*L*(2,1)-labelings of *C*_*i*_⊠*P*_2_ obtained from *V*_*i*,*k*_^3^ in the following way: *x* = *x*_1_ *x*_2_ belongs to *V*(*D*_*i*,*k*_^2^) if and only if there exist *u*, *v* ∈ *V*_*i*,*k*_^3^ such that *u*_1_ = *x*_1_, *u*_2_ = *x*_2_, and *v*_3_ = *x*_1_, *v*_2_ = *x*_2_.

Figure 2(b) shows two vertices of *V*_6,12_^3^ denoted by *u* and *v*. We can see that the labelings of the first and the second copies of *C*_6_ in *u* equal the third and the second copies of *C*_6_ in *v*, respectively. It follows that *V*(*D*_6,12_^2^) possesses the vertex *x* comprising these two labelings.

Let *x*, *y* ∈ *V*(*D*_*i*,*k*_^2^). We make an arc from *x* = *x*_1_ *x*_2_ to *y* = *y*_1_ *y*_2_ if and only if *x*_2_ = *y*_1_ and *x*_1_ *x*_2_ *y*_2_ belongs to *V*_*i*,*k*_^3^.

Note that analogous as above we can improve the method for *P*_*i*_⊠*C*_*ℓ*_. The graph obtained with this procedure (a subgraph of *D*_*i*,*k*_′) will be denoted by *D*_*i*,*k*_^′2^ in the sequel.

For a vertex *v* of a directed graph *D*, the number of inward (resp., outward) directed arcs from *v* in *D* is called an *indegree* (resp., *outdegree*) and denoted by *indeg*_*D*(*v*)_ (resp., *outdeg*_*D*(*v*)_).

We obtain the main result of this section.

**Theorem 9**

*C*_*i*_⊠*C*_*ℓ*_ (resp., *P*_*i*_⊠*C*_*ℓ*_) admits a *k*-*L*(2,1)-labeling if and only if *D*_*i*,*k*_^2^ (resp., *D*_*i*,*k*_^′2^) contains a closed directed walk of length *ℓ*.

**Proof**

It is easy to see that *V*(*D*_*i*,*k*_^2^)⊆*V*(*D*_*i*,*k*_) and *A*(*D*_*i*,*k*_^2^)⊆*A*(*D*_*i*,*k*_). From the definition of *V*_*i*,*k*_^2^ for *x* ∈ *V*(*D*_*i*,*k*_^2^) it follows that *indeg*_*D*_*i*,*k*__(*x*) > 0 and *outdeg*_*D*_*i*,*k*__(*x*) > 0.

Suppose now for *y* ∈ *V*(*D*_*i*,*k*_) that *indeg*_*D*_*i*,*k*__(*y*) > 0 and *outdeg*_*D*_*i*,*k*__(*y*) > 0. Since *V*_*i*,*k*_^3^ contains all *k*-*L*(2,1)-labelings of *C*_*i*_⊠*P*_3_ and *outdeg*_*D*_*i*,*k*__(*y*) > 0, there has to be a vertex *v* ∈ *V*_*i*,*k*_^3^ such that *v*_1_ = *y*_1_ and *v*_2_ = *y*_2_. Moreover, since *indeg*_*D*_*i*,*k*__(*y*) > 0, there has to be a vertex *u* ∈ *V*_*i*,*k*_^3^ such that *u*_2_ = *y*_1_ and *u*_3_ = *y*_2_. It follows that *y* belongs to *V*(*D*_*i*,*k*_^2^). We have therefore proven that *x* ∈ *V*(*D*_*i*,*k*_^2^) if and only if *indeg*_*D*_*i*,*k*__(*x*) > 0 and *outdeg*_*D*_*i*,*k*__(*x*) > 0.

Analogously as above we can show that *xy* is an arc in *D*_*i*,*k*_^2^ if and only if *xy* is an arc in *D*_*i*,*k*_. In other words, we can show that *D*_*i*,*k*_^2^ is isomorphic to subgraph of *D*_*i*,*k*_ induced by *V*_*i*,*k*_^2^. It follows that *D*_*i*,*k*_^2^ contains a closed directed walk of length *ℓ* if and only if *D*_*i*,*k*_ contains a closed directed walk of the same size and the proof for *D*_*i*,*k*_ is settled.

Since the proof for *D*_*i*,*k*_^′2^ is analogous, the proof of the theorem is complete.

The algorithm for generating the graph *D*_*i*,*k*_^2^ is depicted in Algorithm 1 (Procedure CREATE GRAPH).

Note that the number of vertices of *D*_*i*,*k*_^2^ can be much smaller than in *D*_*i*,*k*_. Some examples for *i* and *k* of interest are: |*D*_5,14_^2^ | = 386345700, |*D*_6,11_^2^ | = 12336, and |*D*_8,11_^2^ | = 8157632. However, this reduction is not the only positive effect of the new approach. It is also of a great importance that the running time of CREATE GRAPH is *O*(*n* log⁡ *n*), where *n*, the number of vertices in *V*_*i*,*k*_^3^. In order to see this, note that the running time of an efficient sorting algorithm is also within this time bound. Moreover, this is also the running time of the duration of loop, since a single search in an ordered list with *n* elements requires *O*(log⁡ *n*) time.

The final step of the approach is the search for closed direct walks in *D*_*i*,*k*_^2^. We can find these walks by applying a matrix multiplication of the adjacency matrix of *D*_*i*,*k*_^2^ or a breadth (depth) first search in *D*_*i*,*k*_^2^. Since graphs *D*_*i*,*k*_^2^ are relatively sparse for *i* and *k* of interest, the later approach has been applied in order to compute the results of this paper.

### 3.2. SAT Reduction for *k*-*L*(2,1)-Labeling

The approach is proposed in [17] for the distance-constrained labeling problem. Here we present this method adapted for *L*(2,1)-labeling.

Let *G* = (*V*, *E*) be a graph and *k* a positive integer. For every *v* ∈ *V* and every *i* ∈ {0,1, 2,…, *k*} introduce an atom *x*_*v*,*i*_. Intuitively, this atom shows that the vertex *v* is assigned the color *i*. Consider the following propositional formulas:

1. for all *v* ∈ *V*, ∨_*i*=0_^*k*^ *x*_*v*,*i*_;
2. for all *v* ∈ *V*, 0 ≤ *i* < *j* ≤ *k*, ¬*x*_*v*,*i*_∨¬*x*_*v*,*j*_;
3. for all *v*, *u* ∈ *V*, 0 ≤ *i*, *j* ≤ *k* with *d*(*v*, *u*) = 1 and |*i* − *j* | <2 or *d*(*v*, *u*) = 2 and |*i* − *j* | <1, ¬*x*_*v*,*i*_∨¬*x*_*u*,*j*_.

Clauses (1) and (2) ensure that each vertex is labeled with exactly one label. Clause (3) guarantees that an obtained labeling is a *k*-*L*(2,1)-labeling of *G*. Therefore, the above propositional formulas transform an *L*(2,1)-labeling problem into a propositional satisfiability test (SAT). We can see that an obtained SAT instance is satisfiable if and only if *G* admits a *k*-*L*(2,1)-labeling.

## 4. Results

### 4.1. SAT Reduction

We solve the SAT instances transformed from *L*(2,1)-labeling problems described in Section 4.2 by using the software MiniSat [18]. As a result, we have obtained the *λ*-numbers of *P*_*n*_⊠*C*_*m*_ presented in Table 1 and the *λ*-numbers of *C*_*n*_⊠*C*_*m*_ presented in Table 2.

The values in Table 2 marked with *a* denote the results already obtained in [15], while the entry with 13/14 means that the corresponding value is either 13 or 14.

### 4.2. *λ*-Labeling of *C*_*n*_ ⊠ *C*_*m*_

**Proposition 10**

*λ*(*C*_5_⊠*C*_*m*_) = 14 only if *m* ≡ 0  (mod⁡ 3).

**Proof**

Note that *λ*(*C*_5_⊠*P*_3_) = 14. We can see that *λ*(*C*_5_⊠*P*_3_) ≤ 14 from the fact that every pair of vertices *u*, *v* ∈ *V*(*C*_5_⊠*P*_3_) is at distance at most two. Let *f* denote a 14-*L*(2,1)-labeling of *C*_5_⊠*C*_*m*_ and *f*_*k*_ its restriction to *C*_5_^*k*^. Let also *L*_*k*_ denote the set of labels used in *f*_*k*_. Since *λ*(*C*_5_⊠*P*_3_) = 14, we have |*L*_*k*_ | = 5 and |*L*_*k*_ | +|*L*_*k*+1_ | = 10. Therefore, the restriction of *f* to *C*_5_^*k*+3^ has to comprise the same set of labels as the restriction of *f* to *C*_5_^*k*^ or, more formally, *L*_*k*_ = *L*_*k*+3_. It is straightforward to see that this equality can be satisfied in *C*_5_⊠*C*_*m*_ only if *m* ≡ 0 (mod 3).

**Theorem 11**

Let *m* ≥ 5. Then

(6) $$\begin{array}{c} \lambda \left(C_{5}⊠C_{m}\right)=\{\begin{array}{cc} 14, & m\equiv 0\;\left(\mathrm{mod}\;3\right),\\ 17, & m=7,14,\\ 19, & m=8,\\ 18, & m=11,\\ 16, & m=10,13,17,20,23,26,29,\\ 15, & otherwise\text{.} \end{array} \end{array}$$

**Proof**

Note that the values for *m* ≤ 26 are given in Table 2. We can also show by using the SAT reduction that *λ*(*C*_5_⊠*C*_29_) = 16. Since *λ*(*C*_5_⊠*C*_*j*_) = 14 only if *j* ≡ 0  (mod⁡  3), we construct below a 14-*L*(2,1)-labeling for *C*_5_⊠*C*_3*j*_, *j* ≥ 1, a 15-*L*(2,1)-labeling for *C*_5_⊠*C*_3*j*+1_, *j* ≥ 5, and a 15-*L*(2,1)-labeling for *C*_5_⊠*C*_3*j*+2_, *j* ≥ 10.

Let [*a*, *b*] for *b* ≥ *a* denote the set {*a*, *a* + 1,…, *b*}.

Let *f* denote a function from *V*(*C*_5_⊠*C*_*m*_) to [0,14] and *f*_*k*_ its restriction to *C*_5_^*k*^, *k* ≤ *m* − 1. Let also *L*_*k*_ denote the set of labels used in *f*_*k*_. If we set for *i* ≥ 0 and *s* ∈ {0,1, 2}: *L*_3*i*+*s*_ : = [5*s*, 5*s* + 4], then *f* is a 14-*L*(2,1)-labeling of *C*_5_⊠*C*_3*j*_ for *j* ≥ 1.

Let *f*′ denote a function from *V*(*C*_5_⊠*C*_*m*_) to [0,15] and *f*_*k*_′ its restriction to *C*_5_^*k*^, *k* ≤ *m* − 1. Let also *L*_*k*_′ denote the set of labels used in *f*_*k*_′. If we set for for 0 ≤ *i* ≤ 3: *L*_3*i*_′∶ = [15 − *i*, 15]∪[0,3 − *i*], *L*_3*i*+1_′∶ = [4 − *i*, 8 − *i*], *L*_3*i*+2_′∶ = [9 − *i*, 13 − *i*] and for *i* ≥ 4 and *s* ∈ {0,1, 2}: *L*_3*i*+*s*_∶ = [5*s*, 5*s* + 4], then *f*′ is a 15-*L*(2,1)-labeling of *C*_5_⊠*C*_3*j*+1_ for *j* ≥ 5. As an example, observe the following pattern representing a 15-*L*(2,1)-labeling of *C*_5_⊠*C*_22_:

154914381327121611051005100510051015491438132712161116111611161105101549143813271227122712271216110510154914381338133813381327121611051015491449144914

Let *f*′′ denote a function from *V*(*C*_5_⊠*C*_*m*_) to [0,15] and *f*_*k*_′′ its restriction to *C*_5_^*k*^, *k* ≤ *m* − 1. Let also *L*_*k*_′′ denote the set of labels used in *f*_*k*_′′. If we set for for 0 ≤ *k* ≤ 31: *L*_*k*_′′∶ = *L*_*k*_′ and for *i* ≥ 11 and *s* ∈ {0,1, 2}: *L*_3*i*+*s*_′′∶ = [5*s*, 5*s* + 4], then *f*′′ is a 15-*L*(2,1)-labeling of *C*_5_⊠*C*_3*j*+2_ for *j* ≥ 10. This assertion concludes the proof.

The following results partially depend on comprehensive constructions which provide labelings of interest. These constructions are mostly not included in this paper and can be obtained by the authors.

**Proposition 12**

*λ*(*C*_6_⊠*C*_*m*_) = 11 only if *m* ≡ 0  (mod⁡ 4).

**Proof**

The graph *D*_6,11_^2^ with 12336 vertices and the largest outdegree six has been computed. Since breadth first search algorithm has found only cycles of length four, Theorem 9 yields the proof.

**Theorem 13**

If *m* ≥ 6, then

(7) $$\begin{array}{c} \lambda \left(C_{6}⊠C_{m}\right)=\{\begin{array}{cc} 11, & m\equiv 0\;\left(\mathrm{mod}\;4\right)\\ 16, & m=6\\ 14, & m=7\\ 13, & m=9,10,11,14\\ 12, & otherwise\text{.} \end{array} \end{array}$$

**Proof**

The results for *m* = 6,7, 9,10,11,14 follow from Table 2. We can also see in Table 1 that *λ*(*C*_6_⊠*P*_3_) = 11; thus, from Lemma 1 it follows that *λ*(*C*_6_⊠*C*_*m*_) ≥ 11. Moreover, Proposition 12 says that *λ*(*C*_6_⊠*C*_*m*_) = 11 only if *m* ≡ 0  (mod⁡ 4). In order to see that *λ*(*C*_6_⊠*C*_*m*_) ≤ 12 for other *m* of interest, see as an example a 12-*L*(2,1)-labeling of *C*_6_⊠*C*_13_ depicted in Figure 1.

From Lemma 2 it follows that *λ*(*C*_6_⊠*C*_*m*_) ≤ 12 if *m* ≡ 0  (mod⁡ 13). Analogously, we have found 12-*L*(2,1)-labelings of *C*_6_⊠*C*_40_, *C*_6_⊠*C*_41_, *C*_6_⊠*C*_29_, *C*_6_⊠*C*_30_, *C*_6_⊠*C*_44_, *C*_6_⊠*C*_45_, *C*_6_⊠*C*_33_, *C*_6_⊠*C*_34_, *C*_6_⊠*C*_48_, *C*_6_⊠*C*_36_, *C*_6_⊠*C*_50_, and *C*_6_⊠*C*_38_. Any of these labelings restricted to the first 13 copies of *C*_6_ induces a 12-*L*(2,1)-labeling of *C*_6_⊠*C*_13_. From Lemma 2 it follows that *λ*(*C*_6_⊠*C*_13*k*+1_) ≤ 12 for *k* ≥ 3, *λ*(*C*_6_⊠*C*_13*k*+2_) ≤ 12 for *k* ≥ 3, *λ*(*C*_6_⊠*C*_13*k*+3_) ≤ 12 for *k* ≥ 2, *λ*(*C*_6_⊠*C*_13*k*+4_) ≤ 12 for *k* ≥ 2, *λ*(*C*_6_⊠*C*_13*k*+5_) ≤ 12 for *k* ≥ 3, *λ*(*C*_6_⊠*C*_13*k*+6_) ≤ 12 for *k* ≥ 3, *λ*(*C*_6_⊠*C*_13*k*+7_) ≤ 12 for *k* ≥ 2, *λ*(*C*_6_⊠*C*_13*k*+8_) ≤ 12 for *k* ≥ 2, *λ*(*C*_6_⊠*C*_13*k*+9_) ≤ 12 for *k* ≥ 3, *λ*(*C*_6_⊠*C*_13*k*+10_) ≤ 12 for *k* ≥ 2, *λ*(*C*_6_⊠*C*_13*k*+11_) ≤ 12 for *k* ≥ 3, and *λ*(*C*_6_⊠*C*_13*k*+12_) ≤ 12 for *k* ≥ 2.

Since we have also found 12-*L*(2,1)-labelings of *C*_6_⊠*C*_27_, *C*_6_⊠*C*_28_, *C*_6_⊠*C*_31_, *C*_6_⊠*C*_32_, *C*_6_⊠*C*_35_, and *C*_6_⊠*C*_37_, we conclude that *λ*(*C*_6_⊠*C*_*m*_) ≤ 12 for *m* ≥ 27 and the proof is complete.

**Theorem 14**

If *m* ≥ 7, then

(8) $$\begin{array}{c} \lambda \left(C_{7}⊠C_{m}\right)=\{\begin{array}{cc} 13, & m=7,8,9,14,15,16,17,\\ & \;\;\;18,19,27,28,29\\ 12, & otherwise\text{.} \end{array} \end{array}$$

**Proof**

**Figure 3:**
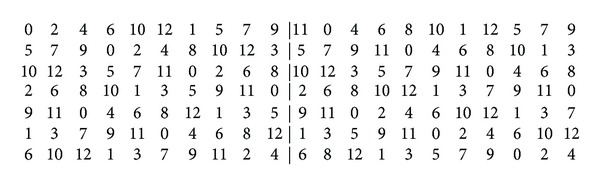
12-*L*(2,1)-labeling of *C*_7_⊠*C*_21_.

The results for *m* ≤ 26 follow from Table 2. We have also established the results for *m* = 27,28,29 by solving the SAT instances transformed from the corresponding *L*(2,1)-labeling problems. Since *λ*(*C*_7_⊠*P*_7_) = 12 as we can see in Table 2, it follows by Lemma 1 that *λ*(*C*_7_⊠*C*_*m*_) ≥ 12 for *m* ≥ 7. In order to see that *λ*(*C*_7_⊠*C*_*m*_) ≤ 12 for other *m* of interest, see as an example a 12-*L*(2,1)-labeling of *C*_7_⊠*C*_21_ depicted in Figure 3. This labeling restricted to the first ten copies of *C*_7_ induces a 12-*L*(2,1)-labeling of *C*_7_⊠*C*_10_. From Lemma 2 it follows that *λ*(*C*_7_⊠*C*_10*k*+1_) ≤ 12, *k* ≥ 2, and *λ*(*C*_7_⊠*C*_10*k*_) ≤ 12, *k* ≥ 1.

Analogously, we have found 12-*L*(2,1)-labelings of *C*_7_⊠*C*_22_, *C*_7_⊠*C*_23_, *C*_7_⊠*C*_34_, *C*_7_⊠*C*_35_, *C*_7_⊠*C*_36_, *C*_7_⊠*C*_47_, *C*_7_⊠*C*_48_, and *C*_7_⊠*C*_49_. Any of these labelings restricted to the first ten copies of *C*_7_ is a 12-*L*(2,1)-labeling of *C*_7_⊠*C*_10_. From Lemma 2 it follows that *λ*(*C*_7_⊠*C*_10*k*+2_) ≤ 12 for *k* ≥ 2, *λ*(*C*_7_⊠*C*_10*k*+3_) ≤ 12 for *k* ≥ 2, *λ*(*C*_7_⊠*C*_10*k*+3_) ≤ 12 for *k* ≥ 2, *λ*(*C*_7_⊠*C*_10*k*+4_) ≤ 12 for *k* ≥ 3, *λ*(*C*_7_⊠*C*_10*k*+5_) ≤ 12 for *k* ≥ 3, *λ*(*C*_7_⊠*C*_10*k*+6_) ≤ 12 for *k* ≥ 3, *λ*(*C*_7_⊠*C*_10*k*+7_) ≤ 12 for *k* ≥ 4, *λ*(*C*_7_⊠*C*_10*k*+8_) ≤ 12 for *k* ≥ 4, and *λ*(*C*_7_⊠*C*_10*k*+9_) ≤ 12 for *k* ≥ 4.

Since we have also found 12-*L*(2,1)-labelings of *C*_7_⊠*C*_27_, *C*_7_⊠*C*_37_, *C*_7_⊠*C*_38_, and *C*_7_⊠*C*_39_, it follows that *λ*(*C*_7_⊠*C*_*m*_) ≤ 12 for all graphs of interest and the proof is complete.

**Proposition 15**

*λ*(*C*_8_⊠*C*_*m*_) = 11 only if *m* ≡ 0  (mod⁡ 6).

**Proof**

The graph *D*_8,11_^2^ with 8157632 vertices and the largest outdegree 8 has been computed. Since breadth first search algorithm has found only cycles of length, six, twelve, and twenty-four, Theorem 9 yields the proof.

**Theorem 16**

If *m* ≥ 8, then

(9) $$\begin{array}{c} \lambda \left(C_{8}⊠C_{m}\right)=\{\begin{array}{cc} 11, & m\equiv 0\;\left(\mathrm{mod}\;6\right)\\ 13, & m=10\\ 12, & otherwise\text{.} \end{array} \end{array}$$

**Proof**

**Figure 4:**
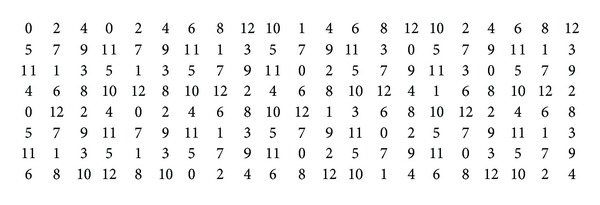
12-*L*(2,1)-labeling of *C*_8_⊠*C*_21_.

Since *λ*(*C*_8_⊠*P*_7_) = 11 (see Table 2), it follows from Lemma 1 that *λ*(*C*_8_⊠*C*_*m*_) ≥ 11.  Proposition 15 says that *λ*(*C*_8_⊠*C*_*m*_) = 11 only if *m* ≡ 0  (mod⁡  6), while the results for *m* ≤ 26 follow from Table 2.  Figure 4 shows a 12-*L*(2,1)-labeling of *C*_8_⊠*C*_21_. This labeling restricted to the first nine copies of *C*_8_ induces a 12-*L*(2,1)-labeling of *C*_8_⊠*C*_9_. From Lemma 2 it follows that *λ*(*C*_8_⊠*C*_9*k*+3_) ≤ 12, *k* ≥ 2, and *λ*(*C*_8_⊠*C*_9*k*_) ≤ 12, *k* ≥ 1.

Moreover, we have found 12-*L*(2,1)-labelings of *C*_8_⊠*C*_46_, *C*_8_⊠*C*_47_, *C*_8_⊠*C*_40_, *C*_8_⊠*C*_50_, *C*_8_⊠*C*_24_, *C*_8_⊠*C*_43_, and *C*_8_⊠*C*_44_. Any of these labelings restricted to the first nine copies of *C*_8_ is a 12-*L*(2,1)-labeling of *C*_8_⊠*C*_9_. From Lemma 2 it follows that *λ*(*C*_8_⊠*C*_9*k*+1_) ≤ 12 for *k* ≥ 5, *λ*(*C*_8_⊠*C*_9*k*+2_) ≤ 12 for *k* ≥ 5, *λ*(*C*_8_⊠*C*_9*k*+4_) ≤ 12 for *k* ≥ 4, *λ*(*C*_8_⊠*C*_9*k*+5_) ≤ 12 for *k* ≥ 5, *λ*(*C*_8_⊠*C*_9*k*+6_) ≤ 12 for *k* ≥ 2, *λ*(*C*_8_⊠*C*_9*k*+7_) ≤ 12 for *k* ≥ 4, and *λ*(*C*_8_⊠*C*_9*k*+8_) ≤ 12 for *k* ≥ 4.

Since we have also found 12-*L*(2,1)-labelings of *C*_8_⊠*C*_28_, *C*_8_⊠*C*_29_, *C*_8_⊠*C*_31_, *C*_8_⊠*C*_32_, *C*_8_⊠*C*_34_, *C*_8_⊠*C*_35_, *C*_8_⊠*C*_37_, *C*_8_⊠*C*_38_, and *C*_8_⊠*C*_41_, we establish the desired upper bound for all graphs of interest and the proof is complete.

**Theorem 17**

If *m* ≥ 9, then

(10) $$\begin{array}{c} \lambda \left(C_{9}⊠C_{m}\right)=\{\begin{array}{cc} 13, & m=9,11,15,14,18,19,22,23\\ 13\;\;or\;\;14, & m=10\\ 12\;\;or\;\;13, & m=27,31,35\\ 12, & otherwise\text{.} \end{array} \end{array}$$

**Proof**

**Figure 5:**
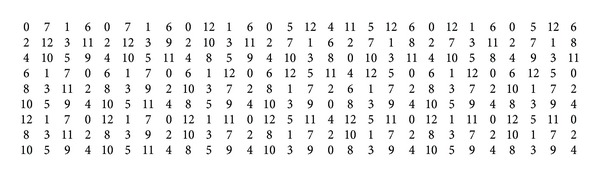
12-*L*(2,1)-labeling of *C*_9_⊠*C*_28_.

Since *λ*(*C*_9_⊠*P*_7_) = 12 (see Table 2), it follows from Lemma 1 that *λ*(*C*_9_⊠*C*_*m*_) ≥ 12. The results for *m* ≤ 26 follow from Table 2. We have also established by solving the SAT instances transformed from the corresponding *L*(2,1)-labeling problems that *λ*(*C*_9_⊠*C*_10_) is either 13 or 14, while for *m* = 27,31,35 the value of *λ*(*C*_9_⊠*C*_*m*_) is either 12 or 13.  Figure 5 shows a 12-*L*(2,1)-labeling of *C*_9_⊠*C*_28_. This labeling restricted to first 12 copies of *C*_9_ induces a 12-*L*(2,1)-labeling of *C*_9_⊠*C*_12_. From Lemma 2 it follows that *λ*(*C*_9_⊠*C*_12*k*+4_) ≤ 12, *k* ≥ 2, and *λ*(*C*_9_⊠*C*_12*k*_) ≤ 12, *k* ≥ 1.

We have also found 12-*L*(2,1)-labelings of *C*_9_⊠*C*_49_, *C*_9_⊠*C*_50_, *C*_9_⊠*C*_63_, *C*_9_⊠*C*_29_, *C*_9_⊠*C*_66_, *C*_9_⊠*C*_55_, *C*_9_⊠*C*_32_, *C*_9_⊠*C*_33_, *C*_9_⊠*C*_58_, and *C*_9_⊠*C*_59_. Any of these labelings restricted to first 12 copies of *C*_9_ induces a 12-*L*(2,1)-labeling of *C*_9_⊠*C*_12_. From Lemma 2 it follows that *λ*(*C*_9_⊠*C*_12*k*+1_) ≤ 12 for *k* ≥ 4, *λ*(*C*_9_⊠*C*_12*k*+2_) ≤ 12 for *k* ≥ 4, *λ*(*C*_9_⊠*C*_12*k*+3_) ≤ 12 for *k* ≥ 5, *λ*(*C*_9_⊠*C*_12*k*+5_) ≤ 12 for *k* ≥ 2, *λ*(*C*_9_⊠*C*_12*k*+6_) ≤ 12 for *k* ≥ 5, *λ*(*C*_9_⊠*C*_12*k*+7_) ≤ 12 for *k* ≥ 4, *λ*(*C*_9_⊠*C*_12*k*+8_) ≤ 12 for *k* ≥ 2, *λ*(*C*_9_⊠*C*_12*k*+9_) ≤ 12 for *k* ≥ 2, *λ*(*C*_9_⊠*C*_12*k*+10_) ≤ 12 for *k* ≥ 4, and *λ*(*C*_9_⊠*C*_12*k*+11_) ≤ 12 for *k* ≥ 4.

Since we have also found 12-*L*(2,1)-labelings of *C*_9_⊠*C*_30_, *C*_9_⊠*C*_34_, *C*_9_⊠*C*_37_, *C*_9_⊠*C*_38_, *C*_9_⊠*C*_39_, *C*_9_⊠*C*_42_, *C*_9_⊠*C*_43_, *C*_9_⊠*C*_46_, *C*_9_⊠*C*_47_, *C*_9_⊠*C*_51_, and *C*_9_⊠*C*_54_, we establish that *λ*(*C*_9_⊠*C*_*m*_) ≤ 12 for all *m* of interest and the proof is complete.

**Theorem 18**

If *m* ≥ 10, then

(11) $$\begin{array}{c} \lambda \left(C_{10}⊠C_{m}\right)=\{\begin{array}{cc} 13, & m=10,11,13,15,16,17,22,26\\ 12\;\;or\;\;13, & 27\leq m\leq 395\\ 12, & otherwise\text{.} \end{array} \end{array}$$

**Proof**

We can see in Table 2 that *λ*(*C*_10_⊠*P*_5_) = 12 hence, it follows by Lemma 1 that *λ*(*C*_10_⊠*C*_*m*_) ≥ 12. The results for *m* ≤ 26 are given in Table 2.

The following pattern represents a 12-*L*(2,1)-labeling of *C*_10_⊠*C*_37_, while the leftmost 12 columns of the pattern represents a 12-*L*(2,1)-labeling of *C*_10_⊠*C*_12_:

831128572011968311210742011968311210574201196 0119631121057420119631121057420119683112105742 7420119683112105720119683112105742011968311210 1121057420119631121057420119683112105742011963 9683112105720119683112105742011968311210572011 4201196311210574201196831121057420119631121057 1210574011948311210574201196831121057201196831 683112857201196831121057420116831121057420119 2011963112105742011968311210742011968311210574 1057401194831121057420116831121057420119683112 

By Lemma 2, we have *λ*(*C*_10_⊠*C*_12*α*+37*β*_) ≤ 12 for integers *α* and *β*. Finally, thanks to Lemma 3, we get *λ*(*C*_10_⊠*C*_*m*_) ≤ 12 for *m* ≥ (37 − 1)·(12 − 1) = 396.

We have found 13-*L*(2,1)-labelings of *C*_10_⊠*C*_*m*_ for 27 ≤ *m* ≤ 46 and we can construct 13-*L*(2,1)-labelings of *C*_10_⊠*C*_*m*_ for *m* ≥ 36 as follows. We have found 13-*L*(2,1)-labelings of *C*_10_⊠*C*_25_, *C*_10_⊠*C*_50_, *C*_10_⊠*C*_27_, *C*_10_⊠*C*_28_, *C*_10_⊠*C*_29_, *C*_10_⊠*C*_54_, and *C*_10_⊠*C*_39_. Any of these labelings restricted to the first eight copies of *C*_10_ is a 13-*L*(2,1)-labeling of *C*_10_⊠*C*_8_. From Lemma 2 it follows that *λ*(*C*_10_⊠*C*_8*k*_) ≤ 13 for *k* ≥ 1, *λ*(*C*_10_⊠*C*_8*k*+1_) ≤ 13 for *k* ≥ 3, *λ*(*C*_10_⊠*C*_8*k*+2_) ≤ 13 for *k* ≥ 6, *λ*(*C*_10_⊠*C*_8*k*+3_) ≤ 13 for *k* ≥ 3, *λ*(*C*_10_⊠*C*_8*k*+4_) ≤ 13 for *k* ≥ 3, *λ*(*C*_10_⊠*C*_8*k*+5_) ≤ 13 for *k* ≥ 3, *λ*(*C*_10_⊠*C*_8*k*+6_) ≤ 13 for *k* ≥ 6, and *λ*(*C*_10_⊠*C*_8*k*+7_) ≤ 13 for *k* ≥ 4. These observations complete the proof.

**Proposition 19**

*λ*(*C*_11_⊠*C*_*m*_) = 10 only if *m* ≡ 0  (mod⁡ 11).

**Proof**

The graph *D*_11,10_^2^ with 380 vertices and the largest outdegree 2 has been computed. Since breadth first search algorithm has found only cycles of length length 11, Theorem 9 yields the proof.

**Theorem 20**

If *m* ≥ 11, then

(12) $$\begin{array}{c} \lambda \left(C_{11}⊠C_{m}\right)=\{\begin{array}{cc} 10, & m\equiv 0\;\left(\mathrm{mod}\;11\right)\\ 12, & m\in \{12,13,14,15,16,18,19,\\ & \;\;\;\;20,21,22,23,25,26\}\\ 11 & m\geq 270\\ 11\;or\;12, & otherwise. \end{array} \end{array}$$

**Proof**

For *m* ∈ {12,13,14,15,16,18,19,20,21,22,23,25,26} the *λ* numbers are obtained by using the SAT reduction as depicted in Table 1. Since *λ*(*C*_11_⊠*P*_3_) = 10, from Lemma 1 it follows that *λ*(*C*_11_⊠*C*_*m*_) ≥ 10, while from Proposition 19 it follows that *λ*(*C*_11_⊠*C*_*m*_) ≥ 11 if *m*≢0 (mod 11).

The result for *m* ≡ 0  (mod⁡ 11) can be obtained by the fact that *λ*(*C*_11_⊠*C*_11_) = 10 and by Lemma 2.

**Figure 6:**
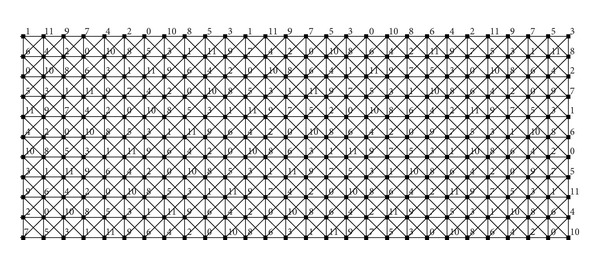
11-*L*(2,1)-labeling of *C*_11_⊠*C*_28_.

Figure 6 represents an 11-*L*(2,1)-labeling of *C*_11_⊠*C*_28_, where the leftmost 11 columns of the figure represent an 11-*L*(2,1)-labeling of *C*_11_⊠*C*_11_. By Lemma 2, we have *λ*(*C*_11_⊠*C*_11*α*+28*β*_) ≤ 11 for integers *α* and *β*. Finally, thanks to Lemma 3, we get *λ*(*C*_11_⊠*C*_*m*_) ≤ 11 for *m* ≥ (28 − 1)·(11 − 1) = 270.

**Figure 7:**
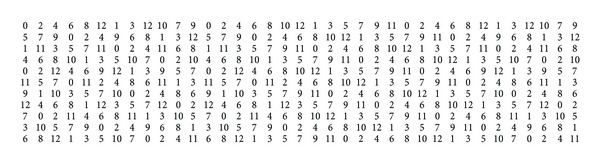
12-*L*(2,1)-labeling of *C*_11_⊠*C*_37_.

In order to find the general upper bound, we present the constructions showing that *λ*(*C*_11_⊠*C*_*m*_) ≤ 12 for *m* ≥ 26. In particular, Figure 7 shows a 12-*L*(2,1)-labeling of *C*_11_⊠*C*_37_. This labeling restricted to the first 12 copies of *C*_11_ induces a 12-*L*(2,1)-labeling of *C*_11_⊠*C*_12_. From Lemma 2 it follows that *λ*(*C*_11_⊠*C*_12*k*+1_) ≤ 12, *k* ≥ 3, and *λ*(*C*_11_⊠*C*_12*k*_) ≤ 12, *k* ≥ 1.

Analogously, we have found 12-*L*(2,1)-labelings of *C*_11_⊠*C*_38_, *C*_11_⊠*C*_39_, *C*_11_⊠*C*_40_, *C*_11_⊠*C*_41_, *C*_11_⊠*C*_42_, *C*_11_⊠*C*_43_, *C*_11_⊠*C*_44_, *C*_11_⊠*C*_45_, *C*_11_⊠*C*_46_, and *C*_11_⊠*C*_35_. Any of these labelings restricted to the first 12 copies of *C*_11_ is a 12-*L*(2,1)-labeling of *C*_11_⊠*C*_12_.

From Lemma 2 it follows that *λ*(*C*_11_⊠*C*_12*k*+2_) ≤ 12 for *k* ≥ 3, *λ*(*C*_11_⊠*C*_12*k*+3_) ≤ 12 for *k* ≥ 3, *λ*(*C*_11_⊠*C*_12*k*+4_) ≤ 12 for *k* ≥ 3, *λ*(*C*_11_⊠*C*_12*k*+5_) ≤ 12 for *k* ≥ 3, *λ*(*C*_11_⊠*C*_12*k*+6_) ≤ 12 for *k* ≥ 3, *λ*(*C*_11_⊠*C*_12*k*+7_) ≤ 12 for *k* ≥ 3, *λ*(*C*_11_⊠*C*_12*k*+8_) ≤ 12 for *k* ≥ 3, *λ*(*C*_11_⊠*C*_12*k*+9_) ≤ 12 for *k* ≥ 3, *λ*(*C*_11_⊠*C*_12*k*+10_) ≤ 12 for *k* ≥ 3, and *λ*(*C*_11_⊠*C*_12*k*+11_) ≤ 12 for *k* ≥ 2.

Since we have also found 12-*L*(2,1)-labelings of *C*_11_⊠*C*_27_, *C*_11_⊠*C*_28_, *C*_11_⊠*C*_29_, *C*_11_⊠*C*_30_, *C*_11_⊠*C*_31_, *C*_11_⊠*C*_32_, *C*_11_⊠*C*_33_, and *C*_11_⊠*C*_34_, we establish that *λ*(*C*_11_⊠*C*_*m*_) ≤ 12 for all *m* ≥ 27 and the proof is complete.

### 4.3. *λ*-Numbers of *P*_*n*_ ⊠ *C*_*m*_

**Proposition 21**

If *m* ≥ 3, then

(13) $$\begin{array}{c} \lambda \left(P_{3}⊠C_{m}\right)=\{\begin{array}{cc} 14, & m=5\\ 11, & m\in \left\{3,4,6,7,8,9,10,13,14,18,19\right\}\\ 10, & otherwise\text{.} \end{array} \end{array}$$

**Proof**

For *m* ≤ 12 the results are obtained by solving the SAT instances transformed from the corresponding *L*(2,1)-labeling problems (see Table 1).

**Figure 8:**
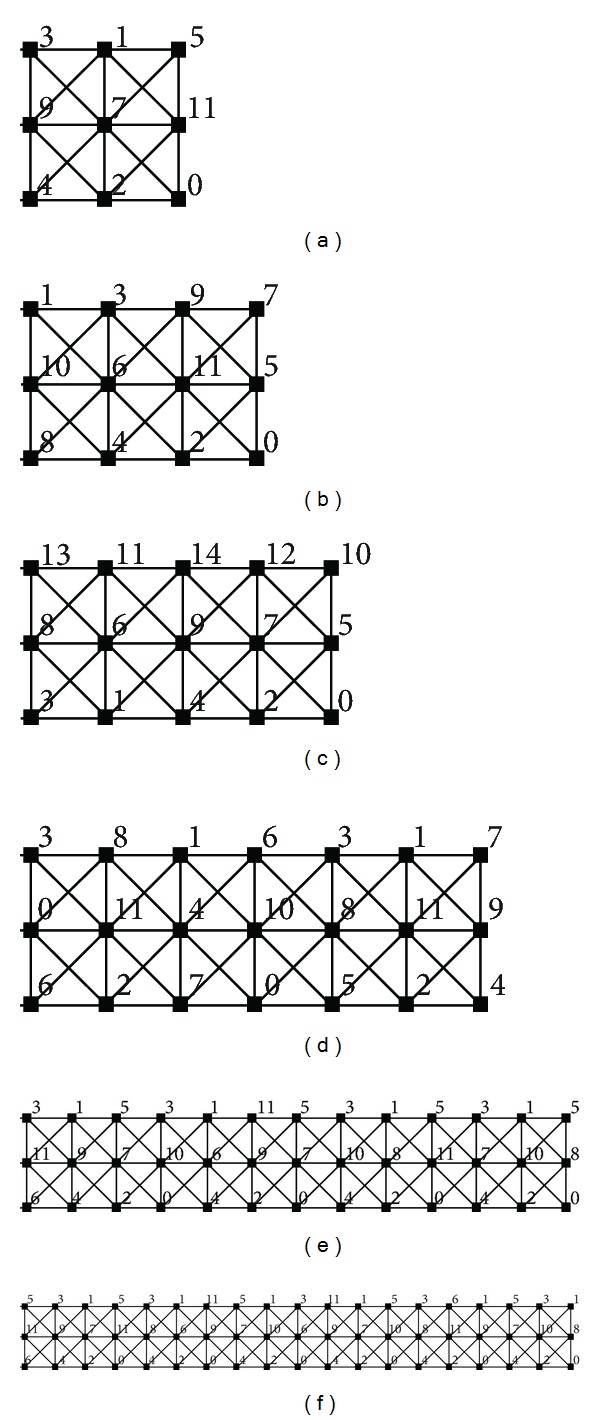
(a) 11-*L*(2,1)-labeling of *P*_3_⊠*C*_3_, (b) 11-*L*(2,1)-labeling of *P*_3_⊠*C*_4_, (c) 14-*L*(2,1)-labeling of *P*_3_⊠*C*_5_, (d) 11-*L*(2,1)-labeling of *P*_3_⊠*C*_7_, (e) 11-*L*(2,1)-labeling of *P*_3_⊠*C*_13_, and (f) 11-*L*(2,1)-labeling of *P*_3_⊠*C*_19_.

The graph *D*_3,10_^′2^ with 9080 vertices and the largest outdegree 16 has been created in order to find 10-*L*(2,1)-labelings in *P*_3_⊠*C*_*m*_. Matrix multiplication has been applied in order to find closed directed walks in the graph. The algorithm has found no closed directed walk of length from the set {3,4, 6,7, 8,9, 10,13,14,18,19}∪{5}. It follows that *λ*(*P*_3_⊠*C*_*n*_) ≥ 11 for any *m* ∈ {3,4, 6,7, 8,9, 10,13,14,18,19}. The upper bounds for *m* ∈ {13,14,18,19} follow from the labelings depicted in Figure 8.

We have found 10-*L*(2,1)-labelings of *P*_3_⊠*C*_23_, *P*_3_⊠*C*_35_, *P*_3_⊠*C*_47_, *P*_3_⊠*C*_26_, *P*_3_⊠*C*_38_, *P*_3_⊠*C*_39_, *P*_3_⊠*C*_40_, *P*_3_⊠*C*_41_, *P*_3_⊠*C*_31_, and *P*_3_⊠*C*_43_. Any of these labelings restricted to the first 11 copies of *P*_3_ is a 10-*L*(2,1)-labeling of *P*_3_⊠*C*_11_. From Lemma 2 it follows that *λ*(*P*_3_⊠*C*_11*k*_) ≤ 10 for *k* ≥ 1, *λ*(*P*_3_⊠*C*_11*k*+1_) ≤ 10 for *k* ≥ 2, *λ*(*P*_3_⊠*C*_11*k*+2_) ≤ 10 for *k* ≥ 3, *λ*(*P*_3_⊠*C*_11*k*+3_) ≤ 10 for *k* ≥ 4, *λ*(*P*_3_⊠*C*_11*k*+4_) ≤ 10 for *k* ≥ 2, *λ*(*P*_3_⊠*C*_11*k*+5_) ≤ 10 for *k* ≥ 3, *λ*(*P*_3_⊠*C*_11*k*+6_) ≤ 10 for *k* ≥ 3, *λ*(*P*_3_⊠*C*_11*k*+7_) ≤ 10 for *k* ≥ 3, *λ*(*P*_3_⊠*C*_11*k*+8_) ≤ 10 for *k* ≥ 3, *λ*(*P*_3_⊠*C*_11*k*+9_) ≤ 10 for *k* ≥ 2, and *λ*(*P*_3_⊠*C*_11*k*+10_) ≤ 10 for *k* ≥ 3.

Since we have also found 10-*L*(2,1)-labelings of *P*_3_⊠*C*_13_, *P*_3_⊠*C*_14_, *P*_3_⊠*C*_15_, *P*_3_⊠*C*_16_, *P*_3_⊠*C*_17_, *P*_3_⊠*C*_18_, *P*_3_⊠*C*_19_, *P*_3_⊠*C*_20_, *P*_3_⊠*C*_21_, *P*_3_⊠*C*_24_, *P*_3_⊠*C*_25_, *P*_3_⊠*C*_27_, *P*_3_⊠*C*_28_, *P*_3_⊠*C*_29_, *P*_3_⊠*C*_30_, *P*_3_⊠*C*_32_, and *P*_3_⊠*C*_36_, we establish that *λ*(*P*_3_⊠*C*_*m*_) ≤ 10 for all *m* ≥ 13 and the proof is complete.

**Proposition 22**

If *m* ≥ 3, then

(14) $$\begin{array}{c} \lambda \left(P_{4}⊠C_{m}\right)=\{\begin{array}{cc} 14, & m=5\\ 10, & m\equiv 0\;\left(\mathrm{mod}\;11\right)\\ 11, & otherwise\text{.} \end{array} \end{array}$$

**Proof**

For *m* = 5 the result is obtained by solving the SAT instance transformed from the corresponding *L*(2,1)-labeling problem. For *m* ≡ 0  (mod⁡ 11) the result follows from Lemma 2 and from the fact that *λ*(*C*_11_⊠*C*_11_) = 10.

In order to find 10-*L*(2,1)-labelings in *P*_4_⊠*C*_*m*_, the graph *D*_4,10_^′2^ with 16792 vertices and the largest outdegree 3 has been created. Since breadth first search algorithm has found only cycles of length 11, the upper bound follows.

For all *m*≢0  (mod⁡ 11) and *m* ≥ 13 we can construct 11-*L*(2,1)-labelings of *P*_4_⊠*C*_*m*_ as described below. We have found 11-*L*(2,1)-labelings of *P*_4_⊠*C*_13_, *P*_4_⊠*C*_14_, *P*_4_⊠*C*_15_, *P*_4_⊠*C*_16_, and *P*_4_⊠*C*_17_. Any of these labelings restricted to the first six copies of *P*_4_ is an 11-*L*(2,1)-labeling of *P*_4_⊠*C*_11_. From Lemma 2 it follows that *λ*(*P*_4_⊠*C*_6*k*+1_) ≤ 11 for *k* ≥ 2, *λ*(*P*_4_⊠*C*_6*k*+2_) ≤ 11 for *k* ≥ 2, *λ*(*P*_4_⊠*C*_6*k*+3_) ≤ 11 for *k* ≥ 2, *λ*(*P*_4_⊠*C*_6*k*+4_) ≤ 11 for *k* ≥ 2, and *λ*(*P*_4_⊠*C*_6*k*+5_) ≤ 11 for *k* ≥ 2. These conclusions complete the proof.

**Corollary 23**

Let *n*, *m* ≥ 4.

- If *m*≢0  (mod⁡ 11), *then λ*(*P*_*n*_⊠*C*_*m*_) ≥ 11,
- If *n*≢0  (mod⁡ 11)  *or m*≢0  (mod⁡ 11), *then λ*(*C*_*n*_⊠*C*_*m*_) ≥ 11.

From Theorem 6 now we have the following

**Corollary 24**

If *m* ≥ 24 and *n* ≥ 26, then

(15) $$\begin{array}{c} \lambda \left(C_{n}⊠C_{m}\right)=\{\begin{array}{cc} 10, & n\equiv 0\;\left(\mathrm{mod}\;11\right)\\ & m\equiv 0\;\left(\mathrm{mod}\;11\right)\\ 11\;or\;12, & otherwise\text{.} \end{array} \end{array}$$

**Proposition 25**

If *m* ≥ 3, then

(16) $$\begin{array}{c} \lambda \left(P_{5}⊠C_{m}\right)=\{\begin{array}{cc} 14, & m=5\\ 12, & m=3,9,10\\ 10, & m\equiv 0\;\left(\mathrm{mod}\;11\right)\\ 11, & m=4,6,7,8,12\;or\;m\geq 80\\ 11\;or\;12, & otherwise\text{.} \end{array} \end{array}$$

**Proof**

For *m* ≤ 12 the results follow from Table 1. For *m* ≡ 0  (mod⁡ 11) the result follows from Lemma 2 and from the fact that *λ*(*C*_11_⊠*C*_11_) = 10.

**Figure 9:**
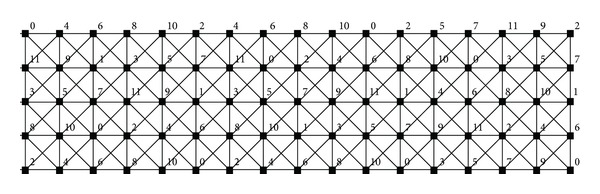
11-*L*(2,1)-labeling of *P*_5_⊠*C*_17_.

Figure 9 represents an 11-*L*(2,1)-labeling of *P*_5_⊠*C*_17_, where the leftmost six columns of the figure represent an 11-*L*(2,1)-labeling of *P*_5_⊠*C*_6_.

By Lemma 2, we have *λ*(*P*_5_⊠*C*_6*α*+17*β*_) ≤ 11 for integers *α* and *β*. Finally, thanks to Lemma 3, we get *λ*(*P*_5_⊠*C*_*m*_) ≤ 11 for *m* ≥ (17 − 1)·(6 − 1) = 80.

In order to complete the proof note that from Theorem 16 and Lemma 1 it follows that *λ*(*P*_5_⊠*C*_*m*_) ≤ 12 for *m* ≥ 11.

**Proposition 26**

If *m* ≥ 3, then

(17) $$\begin{array}{c} \lambda \left(P_{6}⊠C_{m}\right)=\{\begin{array}{cc} 14, & m=5\\ 12, & m=3,9,10\\ 10, & m\equiv 0\;\left(\mathrm{mod}\;11\right)\\ 11, & m=4,6,7,8,12\;or\;m\geq 154\\ 11\;or\;12, & otherwise\text{.} \end{array} \end{array}$$

**Proof**

For *m* ≤ 12 the results follow from Table 1. For *m* ≡ 0  (mod⁡ 11) the result follows Lemma 2 and from the fact that *λ*(*C*_11_⊠*C*_11_) = 10.

**Figure 10:**
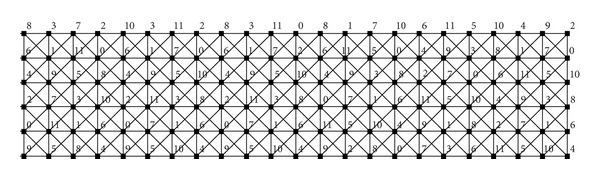
11-*L*(2,1)-labeling of *P*_6_⊠*C*_23_.

Figure 10 represents a 11-*L*(2,1)-labeling of *P*_6_⊠*C*_23_, where the leftmost eight columns of the figure represent an 11-*L*(2,1)-labeling of *P*_6_⊠*C*_8_.

By Lemma 2, we have *λ*(*P*_6_⊠*C*_8*α*+23*β*_) ≤ 11 for integers *α* and *β*. From Lemma 3 it follows that *λ*(*P*_6_⊠*C*_*m*_) ≤ 11 for *m* ≥ (23 − 1)·(8 − 1) = 154.

We complete the proof by noting that from Theorem 16 and Lemma 1 it follows *λ*(*P*_6_⊠*C*_*m*_) ≤ 12 for *m* ≥ 11.

Values in Table 1, the results from Section 4.2, Theorems 6 and 20, and Corollary 23 provide lower and upper bounds for the *λ*-number of *P*_*n*_⊠*C*_*m*_. The results are summarized in the following.

**Theorem 27**

If *n* ≥ 7, then

(18) $$\begin{array}{c} \lambda \left(P_{n}⊠C_{m}\right)=\{\begin{array}{cc} 10, & m\equiv 0\;\left(\mathrm{mod}\;11\right)\\ 14, & m=5\\ 12, & m=3,7,9,10\\ 11, & m=4,6,8,12\;or\;m\geq 270\\ 11\;or\;12, & otherwise. \end{array} \end{array}$$

## 5. Conclusion

In this paper, the *L*(2,1)-labeling problem of the strong product of paths and cycles is studied. The problem derives from the more general Frequency Assignment Problem (FAP) which requires assigning frequencies to transmitters in a wireless network. It is well known that some interesting wireless networks are closely connected to the strong product of graphs. For example, an octagonal grid is the strong product of two paths and an octagonal torus is the strong product of two cycles.

By using various computational approaches, we succeed in solving the problem (except for the final number of cases) for the strong product of a path and a cycle, as well as for the the strong product of two cycles, where one of the cycles is of length at most eleven. Moreover, the obtained results enable us to improve the bounds on the *λ*-number for the strong product of two cycles, where both cycles are sufficiently long. Finding the exact *λ*-numbers for these graphs is therefore an interesting and challenging avenue of further research.

## Figures and Tables

**Algorithm 1:**
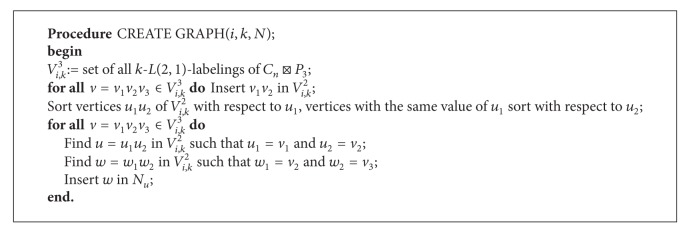


**Table 1:** Values of *λ*(*P*_*n*_  ⊠  *C*_*m*_).

*n*∖*m*	3	4	5	6	7	8	9	10	11	12
3	11	11	14	11	11	11	11	11	10	10
4	11	11	14	11	11	11	11	11	10	11
5	12	11	14	11	11	11	12	12	10	11
6	12	11	14	11	11	11	12	12	10	11
7	12	11	14	11	12	11	12	12	10	11

**Table 2:** Values of *λ*(*C*_*n*_  ⊠  *C*_*m*_).

*n*∖*m*	5	6	7	8	9	10	11	12	13	14	15	16	17	18	19	20	21	22	23	24	25	26
5	24^a^	14^a^	17	19	14	16	18	14	16	17	14	15	16	14	15	16	14	15	16	14	15	16
6		16^a^	14^a^	11	13	13	13	11	12	13	12	11	12	13	12	11	12	12	12	11	12	12
7			13	13	13	12	12	12	12	13	13	13	13	13	13	12	12	12	12	12	12	12
8				13	12	13	12	11	12	12	12	12	12	11	12	12	12	12	12	11	12	12
9					13	13/14	13	12	12	13	13	12	12	13	13	12	12	13	13	12	12	12
10						13	13	12	13	12	13	13	13	12	12	12	12	13	12	12	12	13
11							10	12	12	12	12	12	11	12	12	12	12	10	12	11	12	12
12								11	12	12	12	11	12	11	12	11	12	12	12	11	12	12
